# The protective role of oily fish intake against type 2 diabetes: insights from a genetic correlation and Mendelian randomization study

**DOI:** 10.3389/fnut.2024.1288886

**Published:** 2024-03-19

**Authors:** Youqian Zhang, Entong Ren, Chunlong Zhang, Yang Wang, Xiaohe Chen, Lin Li

**Affiliations:** ^1^Department of Endocrinology, The First Affiliated Hospital of Yangtze University, Jingzhou, Hubei, China; ^2^Health Science Center, Yangtze University, Jingzhou, Hubei, China; ^3^Southern Theater General Hospital, Guangzhou, Guangdong, China; ^4^Department of Nursing, Renmin Hospital of Wuhan University, Wuhan, Hubei, China; ^5^Department of Neurology, The Second Affiliated Hospital of Zhengzhou University, Zhengzhou, China

**Keywords:** Mendelian randomization, oily fish, type 2 diabetes, causal relationship, Linkage Disequilibrium Score

## Abstract

**Background and aims:**

Previous research has underscored the association between oily fish intake and type 2 diabetes (T2DM), yet the causality remains elusive.

**Methods:**

A bidirectional univariable Mendelian Randomization (MR) analysis was employed to evaluate the causal effects of oily fish and non-oily fish intake on T2DM. Replication analysis and meta-analysis were conducted to ensure robust results. Multivariable MR analysis was utilized to assess confounders, and further mediation MR analysis discerned mediating effects. Linkage Disequilibrium Score (LDSC) analysis was undertaken to compute genetic correlations. Inverse variance weighted (IVW) was the primary method, complemented by a series of sensitivity analyses.

**Results:**

The LDSC analysis unveiled a significant genetic correlation between oily fish intake and T2DM (Genetic correlation: -0.102, *p* = 4.43 × 10^−4^). For each standard deviation (SD) increase in genetically predicted oily fish intake, the risk of T2DM was reduced by 38.6% (OR = 0.614, 95% CI 0.504 ~ 0.748, *p* = 1.24 × 10^−6^, False Discovery Rate (FDR) = 3.72 × 10^−6^). The meta-analysis across three data sources highlighted a persistent causal association (OR = 0.728, 95% CI 0.593 ~ 0.895, *p* = 0.003). No other causal effects were identified (all *p* > 0.5, FDR > 0.5). The main outcomes remained consistent in most sensitivity analyses. Both MVMR and mediation MR analyses emphasized the mediating roles of triglycerides (TG), body mass index (BMI), and 25-hydroxyvitamin D (25OHD) levels.

**Conclusion:**

To encapsulate, there’s an inverse association between oily fish intake and T2DM risk, suggesting potential benefits of oily fish intake in T2DM prevention.

## Introduction

Diabetes Mellitus (DM), a leading metabolic disorder marked by chronic hyperglycemia, is on the rise globally (1). Type 2 diabetes mellitus (T2DM) accounts for approximately 90% of all diabetes cases worldwide (2). Current estimates from the World Health Organization (WHO) indicate that over 422 million individuals suffer from diabetes globally, with projections reaching up to 629 million by 2045 (3, 4). The prevalence of diabetes, especially in developing nations, has been on an upward trajectory. This surge poses a considerable direct and indirect financial burden on society (5). Consequently, identifying novel modifiable risk factors for T2DM is crucial to inform clinical management strategies and mitigate the disease’s onset and progression.

Dietary habits emerge as a pivotal modifiable risk determinant, with burgeoning evidence suggesting that functional foods harbor bioactive components correlated with physiological health benefits pertinent to the prevention and management of T2DM (6). Notably, the consumption of oily fish rich in omega-3 fatty acids has consistently piqued interest. Omega-3 fatty acids, particularly eicosapentaenoic acid (EPA) and docosahexaenoic acid (DHA) are renowned for their anti-inflammatory and cardioprotective attributes (7). Their potential roles in glucose metabolism, anti-inflammatory actions, lipid profile amelioration, and insulin sensitivity could shed light on mechanisms underpinning T2DM prevention (7). A population-based prospective study has indicated an association between oily fish consumption and reduced risk of T2DM (8). Yet, a cross-sectional study by Rashmi D Sahay and colleagues posits a direct correlation between oily fish intake and diabetes prevalence in the general populace (9). Furthermore, a meta-analysis amalgamating 13 cohort studies has furnished evidence of a tepid positive relationship between fish and n-3 fatty acid intake and T2DM risk (10). The discordance in research outcomes, coupled with intrinsic limitations of observational studies such as residual confounding and potential reverse causality, obscures the definitive causal relationship between oily fish intake and T2DM pathophysiology.

Mendelian Randomization (MR) operates as an analytical tool, harnessing genetic variants as instrumental variables (IVs) extracted from genome-wide association studies (GWAS) to examine the causal connection between a risk factor (or exposure) and an associated outcome (11). When set against classic observational methods, MR provides an enhanced perspective of the causal impact of the exposure on the outcome by lessening the impact of confounding elements (12). LD Score Regression (LDSC) functions as a strategy to gauge a trait’s heritability, essentially determining the extent of variance within a trait due to genetic influences (13). Additionally, it evaluates the genetic overlap among varied traits based on summary statistics from GWAS (14). Against this backdrop, our study is designed to explore the genetic tendencies linked to oily fish intake concerning T2DM. Moreover, we delve deeper into the potential roles of nine confounders through Multivariable MR (MVMR) and mediation MR frameworks.

## Materials and methods

### Study design

**Figure 1:**
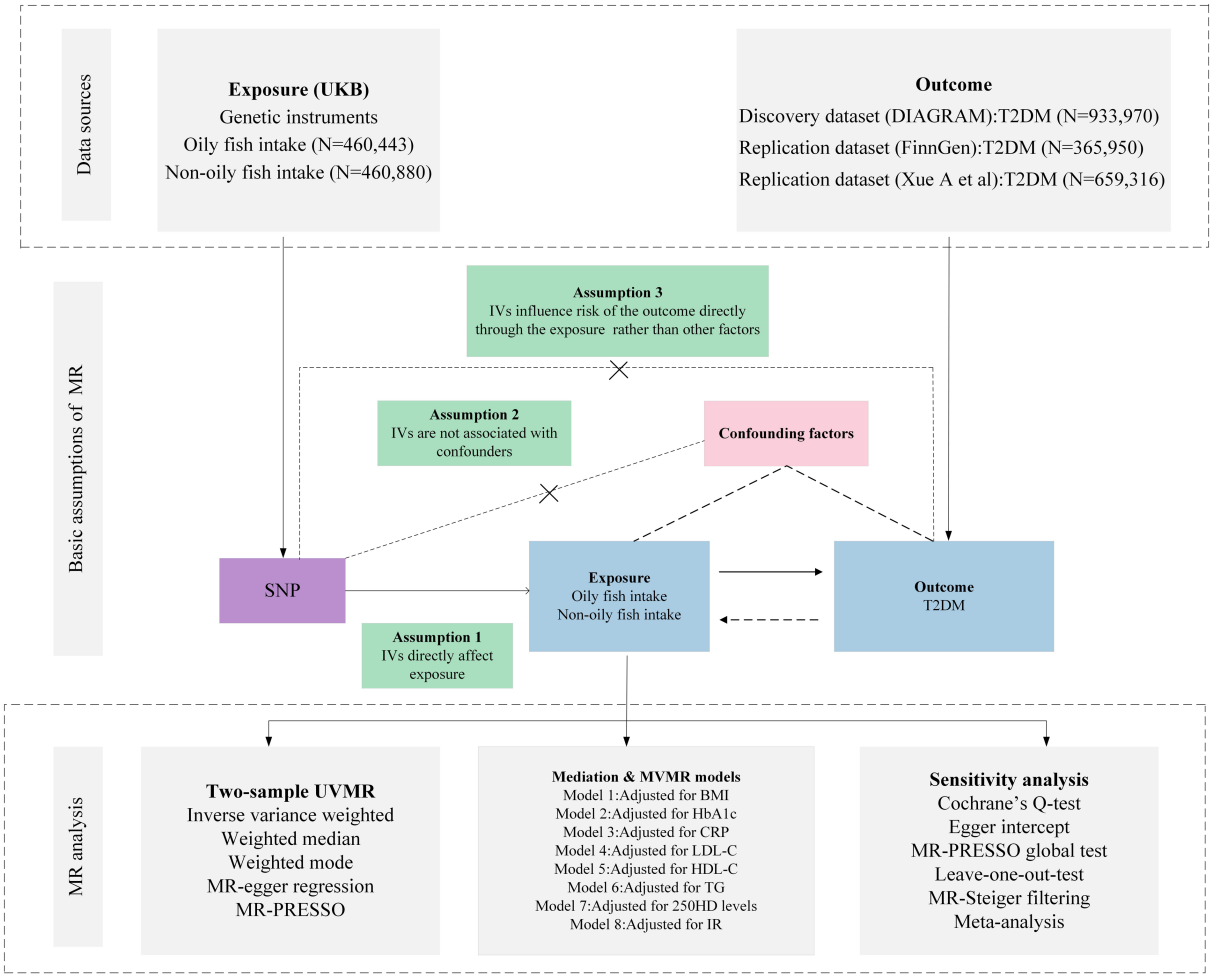
Overview of research design and analysis strategy. Overview of the research design. Exposures come from UKB, with outcomes including T2DM. The MR framework is based on three fundamental MR assumptions, with MVMR analyses adjusting for eight mediating factors for positive results. MVMR: Multivariate Mendelian Randomization; UVMR: Univariate Mendelian Randomization; BMI: Body Mass Index; SNP, Single Nucleotide Polymorphism; MR-PRESSO, MR Pleiotropy Residual Sum and Outlier; HbA1c, Glycated Hemoglobin A1c; LDL-C, Low Density Lipoprotein Cholesterol; HDL-C, High Density Lipoprotein Cholesterol; TG, Triglyceride; T2DM, Type 2 Diabetes Mellitus; CRP, C-Reactive protein; IR, insulin resistance; 25OHD, 25-hydroxyvitamin D; DIAGRAM, DIAbetes Genetics Replication And Meta-analysis; UKB, UK Biobank.

The foundational data for our study was retrieved from open-access, summary-tier datasets stemming from GWAS. We deployed a suite of techniques including Univariate MR (UVMR), MVMR, genetic correlation, mediation MR, and meta-analysis to delve deeply into the causal nexus between the intake of oily and non-oily fish and T2DM. We adopted three pivotal principles in selecting IVs for the exposure: (i) the pinpointed genetic variant, acting as the instrumental variable, demonstrates a robust affiliation with the exposure; (ii) this genetic variant maintains no ties with probable confounders; and (iii) the influence of such variants on the outcome operates exclusively via the exposure, negating the possibility of tangential channels (15). The intricacies of the MR framework can be gleaned from Figure 1. Pertinent details regarding summary statistics data sources are cataloged in Table 1 and the Supplementary File.

**Table 1:** Detailed information of data sources.

Explore or outcome	Ref	Consortium	Ancestry	Participants
Discovery dataset				
Oily fish intake	36,402,876	UKB	European	460,443 individuals
Non-oily fish intake	36,402,876	UKB	European	460,880 individuals
T2DM	35,551,307	DIAGRAM	European	80,154 cases / 853,816 controls
Replication dataset				
T2DM	36,653,562	FinnGen	European	57,698 cases / 308,252 controls
T2DM	30,054,458	Xue et al.	European	62,892 cases / 596,424 controls
Adjustment of the model				
HbA1c	20,858,683	MAGIC	European	46,368 individuals
BMI	30,124,842	GIANT	European	681,275 individuals
TG	32,203,549	Richardson, Tom et al.	European	441,016 individuals
LDL-C	24,097,068	GLGC	96% European	173,082 individuals
HDL-C	24,097,068	GLGC	96% European	187,167 individuals
CRP	30,388,399	Ligthart et al.	European	204,402 individuals
IR	20,081,858	Dupuis et al.	European	37,037 individuals
25OHD levels	32,059,762	Manousaki et al.	European	441,291 individuals

HbA1c, Glycated Hemoglobin A1c; MAGIC, Meta-Analyses of Glucose and Insulin-related traits Consortium; BMI, body mass index; GIANT, Genetic Investigation of Anthropometric Traits; TG, triglycerides; CRP, C-Reactive protein; IR, insulin resistance; LDL-C, Low Density Lipoprotein Cholesterol; HDL-C, High Density Lipoprotein Cholesterol; GLGC, Global Lipids Genetics Consortium; 25OHD, 25-hydroxyvitamin D; DIAGRAM, DIAbetes Genetics Replication And Meta-analysis; T2DM, Type 2 Diabetes Mellitus; UKB, UK Biobank.

All the encompassed GWAS researchers have secured endorsements from their respective institutional review boards. Given that our endeavor pivots on the secondary scrutiny of accessible data, there wasn’t a necessity for further ethical clearance.

### Selection of genetic instrumental variables

The data for oily fish (460,443 individuals) and non-oily fish (460,880 individuals) intake is sourced from the UK Biobank (UKB) Consortium (16). To ensure the accuracy of the MR estimates, these SNPs were required to satisfy specific criteria:

1. All selected Single Nucleotide Polymorphism (SNP)s, when considered as IVs, demonstrated an association with the specific exposure at a genome-wide significance threshold (*p* < 5 × 10^−8^).
2. For the prevention of biases from linkage disequilibrium, the SNPs were ensured not to be associated with any potential confounders and to be independent of each other (*r*^2^ < 0.001, clumping distance = 10,000 kb).
3. The strength of instrumental variables was evaluated using F-statistics, defined by the formula: F = beta^2^/se^2^ (where beta represents the SNP-exposure association and variance is denoted by se). A higher F-statistic signified enhanced instrumental strength, with a mandatory requirement for all SNPs to have an F-statistic exceeding 10 (17).
4. MR-Steiger filtering was integrated to reinforce the validity of our results, thereby omitting variants that had more substantial associations with outcomes than with exposures (18).
5. If the outcome dataset lacks SNP, this study will not use proxy SNPs (*r*^2^ > 0.8) to ensure the accuracy of the outcomes.
6. It was essential that the effect of an SNP on exposure and its effect on the outcome both aligned with the identical allele. SNPs not meeting this alignment were excluded.

### Source of outcome phenotypes

The summary-level GWAS meta-analysis for T2DM (80,154 cases and 853,816 controls) integrated 32 cohorts, sourced from the AMP-T2D Knowledge Portal and the DIAbetes Genetics Replication And Meta-analysis (DIAGRAM) consortium (19). Replication analysis and meta-analysis data for T2DM were sourced from the FinnGen (R9) consortium (20, 57,698 cases and 308,252 controls) (20) and the GWAS by Xue et al. (21). (62,892 cases and 596,424 controls), respectively.

### Data sources for possible mediators

We further obtained genetic associations for Body Mass Index (BMI) from the Genetic Investigation of Anthropometric Traits (GIANT) consortium (22), Glycated Hemoglobin A1c (HbA1c) from Meta-Analyses of Glucose and Insulin-related traits Consortium (MAGIC) (23), triglycerides (TG) from Richardson, Tom et al. (24), Low-Density Lipoprotein Cholesterol (LDL-C) and High-Density Lipoprotein Cholesterol (HDL-C) from Global Lipids Genetics Consortium (GLGC) (25), C-Reactive protein (CRP) from Ligthart et al. (26), insulin resistance (IR) from Dupuis et al. (27), 25-hydroxyvitamin D (25OHD) levels from Manousaki et al. (28).

## Statistical analyses

### Primary MR analysis

In the UVMR analysis, we applied the Wald ratio test to individual IVs. For assessing multiple IVs (≥2), we employed the multiplicative random-effects inverse-variance-weighted (IVW) method, supplemented by MR-Egger and weighted median methods. The weighting in IVW is directly proportional to the Wald ratio estimate and inversely proportional to its variance for each SNP (29). While IVW provides reliable estimates when all genetic variations are valid, the weighted median is more suitable when over half are invalid, and MR-Egger when all are invalid (30). We adjusted for multiple comparisons using the False Discovery Rate (FDR), with a *p*-value <0.05 post-adjustment denoting a significant causal link. If an unadjusted *p*-value was <0.05 but the FDR-adjusted *p*-value was >0.05, it was only suggestive.

For the Mediation MR & MVMR analysis, given potential confounding by factors like BMI, HbA1c, TG, LDL-C, HDL-C, CRP, IR, and 25OHD in the exposure-outcome pathway, we proceeded with MVMR to gauge the direct causal effect. The first MVMR assumption pertains to genetic variation’s link with one or more exposures, while subsequent assumptions mirror those of UVMR (31). We also assessed the proportion of mediated effects. Initially, MR effect estimates for exposure-associated (Oily fish intake) outcomes were derived using IVW. This was followed by MVMR to measure the impact of the eight mediating factors on the outcome. Multiplying the two estimates for each outcome gave the indirect effect of the exposure. By dividing the mediated effect by the total effect, we could determine the contribution of each mediator to the overall effect.

### Genetic correlation analysis

Linkage Disequilibrium Score (LDSC) regression, optimized for summary-level GWAS datasets, provides an advanced methodology to analyze genetic correlations in multifaceted diseases or phenotypic traits. This method adeptly differentiates true polygenic associations from confounders like cryptic relatedness or population stratification (32). If a genetic correlation is both statistically and quantitatively significant, it suggests that the overall phenotype correlation is not solely driven by environmental confounding factors (32). The LDSC platform, accessible at https://github.com/bulik/ldsc, serves as a tool for investigating genetic correlations between exposures and their associated outcome phenotypes.

### Sensitivity analysis

In the UVMR framework, a battery of tests was conducted to ensure analytical rigor. Cochran’s Q test was employed to gauge the heterogeneity across the selected genetic markers, with a *p*-value below 0.05 indicating significant discrepancies among the investigated SNPs (33). The potential for directional pleiotropy within the MR schema was assessed via MR-Egger regression (34). An MR-Egger’s intercept *p*-value under 0.05 suggests notable directional pleiotropy, though this approach may have relative limitations in precision (35). To identify outliers and evaluate horizontal pleiotropy, the MR Pleiotropy Residual Sum and Outlier (MR-PRESSO) technique was adopted, with significance inferred when the global *p*-value is less than 0.05 (36). By excluding outliers, this method aids in refining our corrections. The leave-one-out technique was also applied to determine the impact of singular SNPs on the cumulative findings (37).

The R^2^ value was determined using the equation 2 × MAF×(1-MAF) × beta^2^, where MAF stands for the minor allele frequency for each instrumental SNP. The resulting values were then aggregated to obtain the coefficient essential for the power calculation (38). The statistical power was calculated based on the mRnd website (39).^1^

## Results

### Genetic instrument selection and genetic correlation between phenotypes

The study reports F statistics exceeding 40 for all instrumental variants, signifying a robust reduction of bias from weak instruments. The SNPs selected as IVs ranged from 9 to 177, accounting for an explained variance of 0.05 to 27.29%, and the power statistics obtained ranged from 14 to 100% (Supplementary Table S1).

LDSC analysis revealed a significant genetic correlation between oily fish intake and T2DM (r_g_: -1.102, *p* = 4.43 × 10^−4^), but no genetic association was found with non-oily fish intake (r_g_: 0.024, *p* = 0.477). The SNP-based liability-scale heritability (h^2^) spanned from 2.49 to 5.76% (Supplementary Table S2).

### Association of genetically predicted oily fish and non-oily fish intake with T2DM

**Figure 2:**
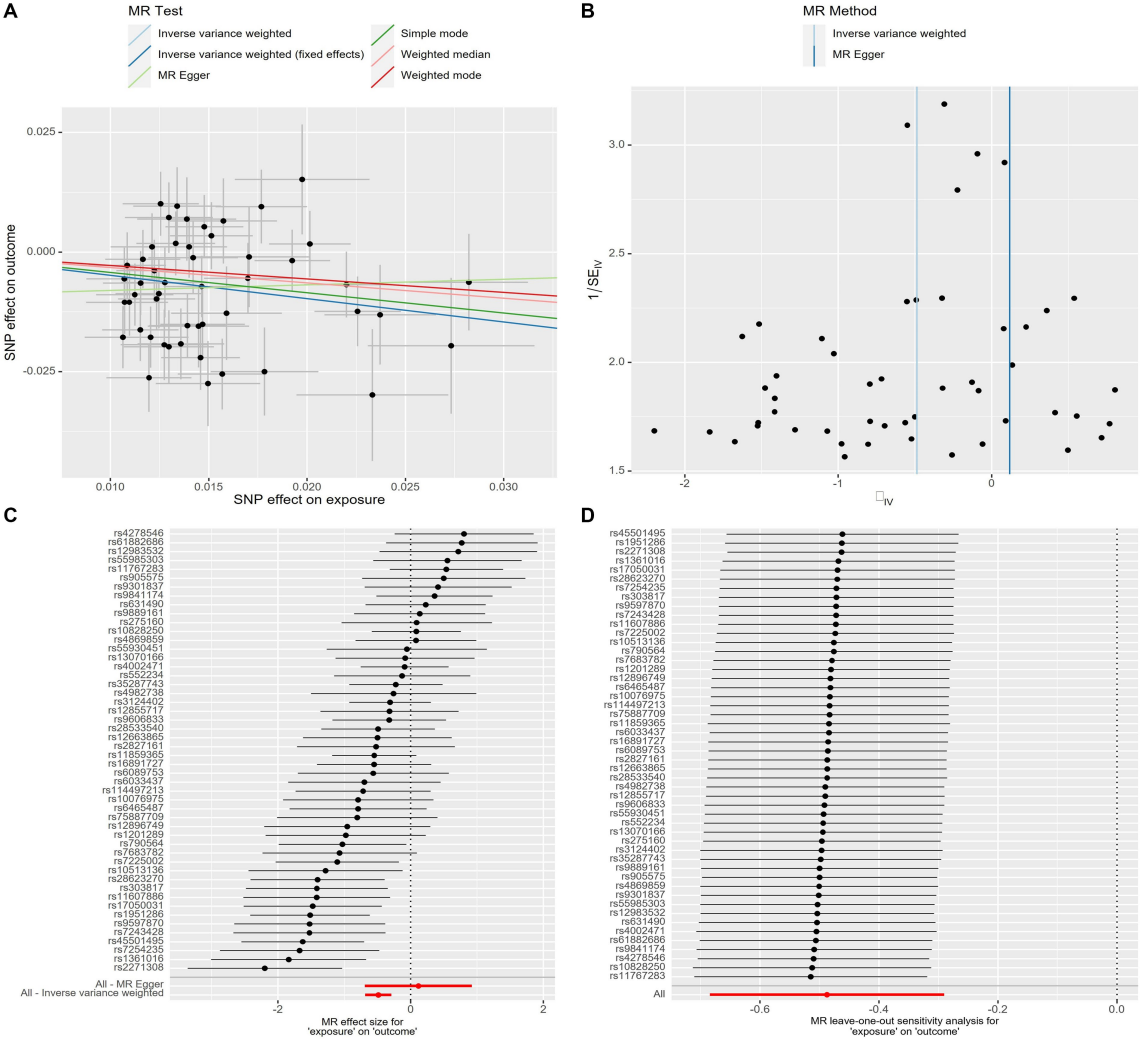
Genetic associations with oily fish intake with T2DM at a genome-wide level of significance. **(A)** Scatter plot. **(B)** Funnel plot. **(C)** Forest plots. **(D)** Leave-one-out plot. Horizontal and vertical lines represent 95% confidence intervals for the genetic associations. T2DM, T2DM, Type 2 Diabetes Mellitus.

**Figure 3:**
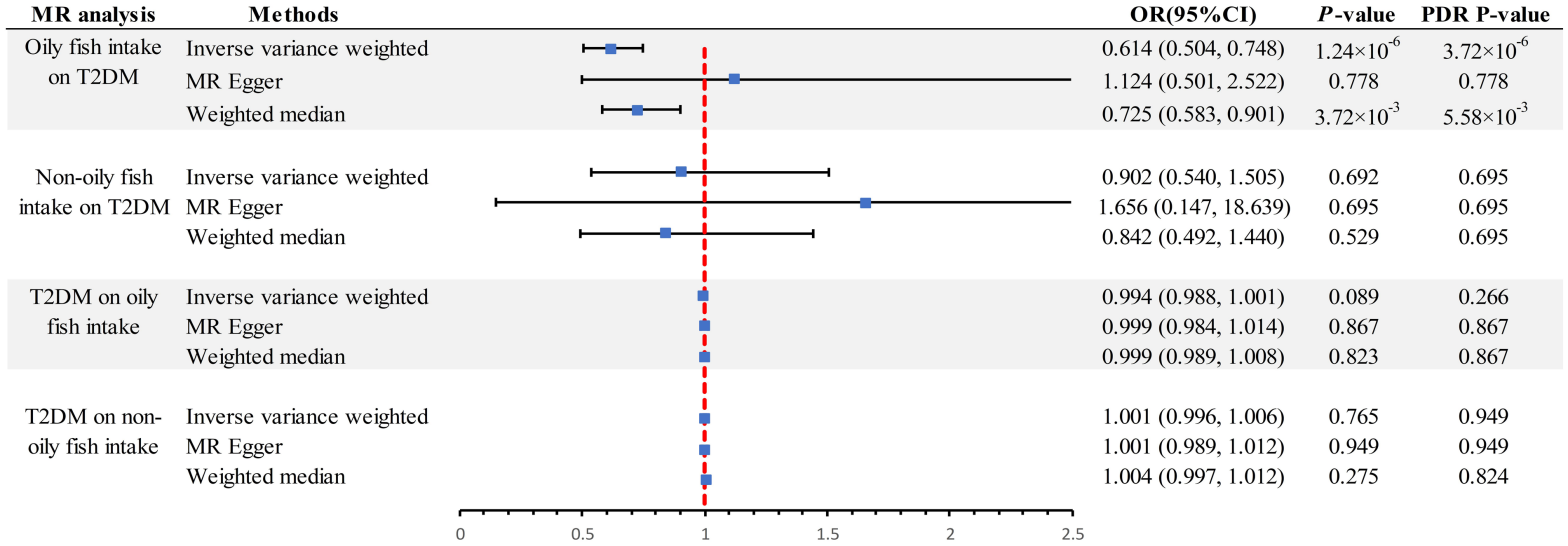
Summary of estimates of genetically predicted oily fish and non-oily fish intake for T2DM. FDR, False Discovery Rate; OR odds ratio; CI, confidence interval; T2DM, Type 2 diabetes mellitus; MR, Mendelian randomization.

The scatter plot provides a clear visualization of all positive results (Figure 2). After applying the FDR correction for multiple comparisons, our primary IVW analysis demonstrated strong causal evidence (Figure 3). Specifically, for every standard deviation (SD) increase in genetically predicted oily fish intake, the risk of T2DM reduced by 38.6% (OR = 0.614, 95% CI 0.504 ~ 0.748, *p* = 1.24 × 10^−6^, FDR = 3.72 × 10^−6^). Additionally, when the OR was 0.614, we possessed a 100% Power-statistic to detect a correlation between oily fish intake and T2DM. No causal relationship was found between non-oily fish intake and T2DM (OR = 0.902, 95% CI 0.540 ~ 1.505, *p* = 0.692, FDR = 0.695). In further reverse MR analysis, no causal relationship was observed as well (all *p* > 0.05, FDR >0.05) (Supplementary Table S3).

A series of sensitivity analyses confirmed the robustness of the UVMR results (Supplementary Table S4). MR-Egger detected no horizontal pleiotropy (*p* > 0.05). The leave-one-out analysis further validated that the causal relationship wasn’t influenced by any single SNP, and the funnel plot showed symmetry (Figure 2; Supplementary Figures S1–S3). The Steiger test indicated that all SNPs passed the test, and the direction of causality remained unchanged, further solidifying the results.

### Meta-analysis of oily fish intake’s effect on T2DM across three data sources

**Figure 4:**
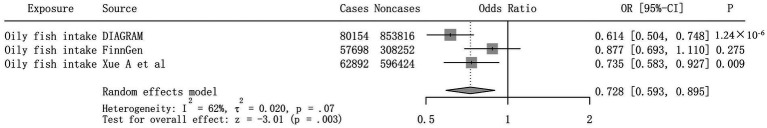
Mendelian randomization association of genetically predicted oily fish intake with T2DM in meta-analysis. Odds ratios are scaled per SD increase in the genetically predicted oily fish intake. OR, odds ratio; CI, confidence interval; T2DM, type 2 diabetes.

To assess the consistency of the causal association between oily fish intake and T2DM across different data sources, primary analyses were replicated based on the FinnGen consortium and the GWAS by Xue et al. Using the GWAS data from Xue et al., the causal effect of oily fish intake on T2DM remained evident (OR = 0.735, 95% CI 0.583 ~ 0.927, *p* = 0.009); however, the causal effect was no longer significant in the FinnGen consortium data. In the replication analyses of FinnGen Consortium and Xue et al., we had 45 and 95% Power-statistic detection of an association between oily fish intake and T2DM, with ORs of 0.877 and 0.735, respectively (Supplementary Table S1). A series of sensitivity analyses confirmed the robustness of the results. Ultimately, a meta-analysis aggregating three distinct data sources provided consistent evidence of a causal link between oily fish intake and T2DM (OR = 0.728, 95% CI 0.593 ~ 0.895, *p* = 0.003) (Figure 4).

### Mediation & MVMR analysis

In the MVMR analysis, only outcome phenotypes with strong associations were selected for multivariable analysis. The MR estimates for oily fish intake and T2DM lost statistical significance after adjusting for BMI, HbA1c, CRP, IR, and 250HD levels, indicating the influence of confounding factors (Table 2). Further mediation MR analysis revealed that BMI mediated the primary mediating effect in the causal association between oily fish and T2DM (mediation effect: 33.57%). Additionally, TG (mediation effect: 8.55%) and 25OHD levels (mediation effect: 11.65%) played roles in partial mediation (Table 3). The causal relationships among the three conformed to the principles of mediation MR.

**Table 2:** Multivariable Mendelian randomization associations of oily fish intake with T2DM adjusting for confounding factors.

Model	SNPs	Direct effect	OR	95%Cl	*p*-value
Adjusted for BMI	474	0.074	1.076	(0.684,1.694)	0.750
Adjusted for HbA1c	225	−0.085	0.918	(0.610, 1.381)	0.682
Adjusted for CRP	71	−0.195	0.823	(0.330, 2.052)	0.676
Adjusted for IR	25	−0.427	0.653	(0.285, 1.497)	0.314
Adjusted for 25OHD levels	181	−0.182	0.834	(0.576, 1.207)	0.335
Adjusted for TG	292	−0.582	0.559	(0.344, 0.908)	0.019
Adjusted for LDL-C	92	−0.598	0.550	(0.310, 0.977)	0.041
Adjusted for HDL-C	103	−0.887	0.412	(0.219, 0.775)	0.006

The effect of potential confounders was adjusted individually. BMI, body mass index; OR, odds ratio; CI, confidence interval; HbA1c, Glycated Hemoglobin A1c; TG, triglycerides; CRP, C-Reactive protein; IR, insulin resistance; LDL-C, Low Density Lipoprotein Cholesterol; HDL-C, High Density Lipoprotein Cholesterol; 25OHD, 25-hydroxyvitamin D.

**Table 3:** Mediation analysis of the mediation effect of oily fish intake on T2DM via eight confounding factors

Exposure	Mediator	Total effect Effect size (95% CI)	Direct effect Effect size (95% CI)	Mediation effect		
				Effect size (95% CI)	IE div TE(%)	*p*
Oily fish intake	HbA1c	−0.393(−0.629,−0.157)	−0.380(−0.616,−0.144)	−0.013(−0.028,0.002)	3.35%	0.080
	BMI	−0.393(−0.628,−0.157)	−0.261(−0.521,−0.001)	−0.132(−0.242,−0.022)	33.57%	0.019
	TG	−0.342(−0.578,−0.105)	−0.313(−0.550,−0.075)	−0.342(−0.579,−0.105)	8.55%	0.001
	LDL-C	−0.393(−0.629,−0.157)	−0.393(−0.629,−0.158)	0.000(−0.007,0.008)	−0.08%	0.935
	HDL-C	−0.393(−0.628,−0.157)	−0.387(−0.623,−0.151)	−0.006(−0.021,0.008)	1.55%	0.378
	CRP	−0.393(−0.629,−0.157)	−0.392(−0.629,−0.156)	0.000(−0.021,0.020)	0.10%	0.970
	IR	−0.393(−0.629,−0.157)	−0.393(−0.630,−0.157)	0.000(−0.019,0.020)	−0.11%	0.901
	25OHD levels	−0.342(−0.578,−0.105)	−0.302(−0.540,−0.063)	−0.039(−0.068,−0.012)	11.65%	0.005

HbA1c, Glycated Hemoglobin A1c; BMI, body mass index; TG, triglycerides; CRP, C-Reactive protein; IR, insulin resistance; LDL-C, Low Density Lipoprotein Cholesterol; HDL-C, High Density Lipoprotein Cholesterol; 25OHD, 25-hydroxyvitamin D; IE div TE, Indirect Effect divided by Total Effect; OR, odds ratio; CI, confidence interval.

## Discussion

This study utilized a comprehensive MR analysis to investigate the relationship between genetic susceptibility related to oily and non-oily fish intake and T2DM. MR results support previous epidemiological studies (8), suggesting a causal relationship between oily fish consumption and a decreased T2DM risk. Such associations were consistent across replication and meta-analyses. MVMR analysis highlighted the impact of various confounders. Mediation MR revealed that BMI mediated a third of the effect, while TG and 250HD contributed further. The study will delve into the significance of these findings from various perspectives, including the roles of inflammatory and oxidative stress biomarkers, homocysteine metabolism, Omega-3 fatty acids, and mediating factors.

Oily fish, a rich source of omega-3 fatty acids like EPA and DHA, plays a significant role in anti-inflammatory processes. EPA and DHA mitigate inflammation by inhibiting the production of pro-inflammatory eicosanoids derived from arachidonic acid. A double-blind study by Jisun So and colleagues highlights that while DHA exhibits stronger anti-inflammatory effects, EPA notably balances pro-inflammatory and anti-inflammatory proteins (40). Additionally, both EPA and DHA downregulate the expression of inflammatory cytokines. *In vitro* and animal studies demonstrate their effectiveness in reducing the release of tumor necrosis factor-alpha (TNF-α), interleukin-6 (IL-6), and interferon gamma-induced protein 10 (IP-10), cytokines often overexpressed in T2DM patients (41, 42). Extensive research indicates that chronic inflammation accelerates oxidative stress, leading to cellular and tissue damage, including in pancreatic β-cells (43, 44). Omega-3 fatty acids can lower reactive oxygen species (ROS) levels and enhance the activity of antioxidant enzymes such as superoxide dismutase (SOD) and glutathione peroxidase, pivotal in neutralizing harmful free radicals and maintaining cellular balance. This reduction in oxidative stress damage, in line with Marilena Lepretti’s findings (45), underscores the potential mechanism behind oily fish consumption’s protective role against T2DM.

Elevated homocysteine levels are considered a risk factor for T2DM, with Tao Huang’s MR and meta-analysis establishing a causal relationship (46). Homocysteine is metabolized through two primary pathways: remethylation and transsulfuration. Remethylation involves converting homocysteine back to methionine, a process critically dependent on vitamins B6, B12, and folate. Jie Zhu’s research suggests that a combination of omega-3 fatty acids, folate, and vitamins B6 and B12 is more effective in reducing plasma homocysteine levels (47), with omega-3 fatty acids indirectly supporting this pathway by maintaining healthy levels of these vitamins. Conversely, transsulfuration converts homocysteine to cysteine, which is further used in glutathione synthesis, an effective antioxidant (48). Omega-3 fatty acids enhance the transsulfuration pathway, reducing homocysteine levels and bolstering antioxidant defenses. Additionally, Sohrab A. Shaikh’s review links elevated homocysteine levels with IR (49), where omega-3’s anti-inflammatory and metabolic regulatory functions can mitigate resistance. Carlos O Mendivil’s study also highlights the role of oily fish consumption in regulating homocysteine levels (50). Thus, given the association between homocysteine levels and T2DM, emphasizing oily fish intake is crucial in T2DM management.

While observational studies have inherent limitations, such as potential confounding factors and the challenge of distinguishing causation from correlation, the use of MR analysis can help overcome these biases, aiming to identify genuine causal associations. The omega-3 fatty acids in oily fish have gained significant attention for their possible protective effects against diabetes. Marilena Lepretti’s research suggests that the presence of EPA and DHA in cell membranes might enhance insulin receptor function (45). This is believed to be due to these fatty acids increasing membrane fluidity, which facilitates the insulin receptor’s movement and its interaction with insulin. Silvia V. Conde’s study further supports this, demonstrating that a higher omega-3 intake can enhance insulin signaling and sensitivity (51). Apart from omega-3 s, other nutrients in oily fish, like proteins and fats, have essential roles in blood glucose stabilization. A study from the Joslin Diabetes Center found that a combination of proteins and fats could slow carbohydrate digestion and its absorption, maintaining steady glucose levels (52). Regular fluctuations in blood glucose can lead to increased insulin secretion, which, over time, might result in IR.

Omega-3 fatty acids are also known for their anti-inflammatory properties. They can reduce pro-inflammatory cytokine production, such as tumor necrosis factor-α and interleukin-6, potentially reducing IR (53). Afiat Berbudi’s comprehensive review highlights the importance of these anti-inflammatory effects in decreasing T2DM risk (54). In the context of lipid metabolism, A Jaca’s systematic review indicates that omega-3 s can significantly lower triglyceride levels, possibly by inhibiting Very low-density lipoproteins (VLDL) synthesis and secretion (55). Minglei Ma’s research further clarifies this, suggesting that reduced triglyceride levels are linked to increased insulin sensitivity (56). This provides more context to the mediator MR’s findings about the effects of TG and BMI. Oily fish stands as a substantial source of vitamin D; increased consumption can potentially raise plasma levels of 25-hydroxyvitamin D. On the role of vitamin D, a pivotal randomized controlled trial (RCT) by Anastassios G Pittas and his team delineated the consequential role of vitamin D in the prevention of T2DM. It was found that vitamin D can augment the function of pancreatic β-cells, thereby increasing insulin secretion. Additionally, it can potentiate the action of insulin, resulting in a reduction of blood glucose levels (57).

The study has several strengths. First, our MR analysis novelly establishes the causal relationship between oily fish consumption and T2DM, which is further supported by consistent meta-analysis evidence. Second, the exclusive identification of SNPs as IVs within European populations reduces potential population stratification biases. Third, our rigorous methodologies, paired with F-statistics exceeding 10, minimize potential biases from weak instruments. Fourth, the influence of confounders was examined through MVMR, and mediation MR further elaborated the roles of BMI, TG, and 250HD. Fifth, we included varied statistical model-based sensitivity analyses and “leave-one-out” techniques to bolster our findings’ reliability. However, our study is not without limitations. Notably, while MR-PRESSO analysis attempted to eliminate horizontal pleiotropy, we could not fully exclude it, but supplementary sensitivity evaluations reaffirm our results’ robustness. Also, our dependence on summary-level GWAS data means subgroup analyses were not possible.

## Conclusion

To encapsulate, there’s an inverse association between oily fish intake and T2DM risk, suggesting the potential benefits of oily fish intake in T2DM prevention.

## Data availability statement

The original contributions presented in the study are included in the article/Supplementary material, further inquiries can be directed to the corresponding author.

## Ethics statement

Ethical approval was not required for the studies involving humans because all the encompassed GWAS researchers have secured endorsements from their respective institutional review boards. Given that our endeavor pivots on the secondary scrutiny of accessible data, there wasn’t a necessity for further ethical clearance. The studies were conducted in accordance with the local legislation and institutional requirements. The participants provided their written informed consent to participate in this study.

## Author contributions

YZ: Writing – original draft, Writing – review & editing. ER: Investigation, Supervision, Writing – review & editing. CZ: Validation, Writing – review & editing. YW: Writing – review & editing. XC: Writing – review & editing. LL: Writing – review & editing.

## Footnotes

^1^ https://shiny.cnsgenomics.com/mRnd/
