# Supplementary material for: The protective role of oily fish intake against type 2 diabetes: insights from a genetic correlation and Mendelian randomization study
Source: Front Nutr. 2024 Mar 19;11:1288886. doi: 10.3389/fnut.2024.1288886 (PMC10986736; doi:10.3389/fnut.2024.1288886)
Supplement: Supplementary file 1 [file Data_Sheet_1.docx]

**Description of summary statistics data sources**

**GWAS of Type 2 Diabetes Mellitus (T2DM) by DIAGRAM (DIAbetes Genetics Replication And Meta-analysis) consortium**^1^**.**

The data sources for T2DM GWAS summary statistics include the AMP-T2D Knowledge Portal and the DIAGRAM Consortium Data Download website. The AMP-T2D Knowledge Portal provides association summary statistics from the multi-ancestry meta-analysis and annotation-informed fine-mapping. The DIAGRAM Consortium Data Download website provides summary statistics from the largest T2DM GWAS meta-analysis to date, which includes data from over 100,000 individuals of European, South Asian, East Asian, and African ancestry.

The multi-ancestry meta-analysis identified 237 loci attaining stringent genome-wide significance (*P* < 5×10^-9^), which were delineated to 338 distinct association signals. Fine-mapping of these signals was enhanced by the increased sample size and expanded population diversity of the multi-ancestry meta-analysis, which localized 54.4% of T2DM associations to a single variant with >50% posterior probability. This improved fine-mapping enabled systematic assessment of candidate causal genes and molecular mechanisms through which T2DM associations are mediated, laying the foundations for functional investigations.

The multi-ancestry genetic risk scores enhanced transferability of T2DM prediction across diverse populations. The study also identified several novel loci associated with T2DM, including those involved in insulin secretion and beta-cell function, as well as those involved in lipid metabolism and inflammation. In addition to these primary data sources, there are also several consortia that have contributed to T2DM GWAS. The FinnGen consortium has contributed to T2DM GWAS by conducting genome-wide association analyses in a Finnish population-based cohort of over 135,000 individuals. The eMERGE consortium has contributed to T2DM GWAS by conducting genome-wide association analyses in electronic medical record-linked biobanks from over 50,000 individuals of diverse ancestry.

The summary statistics data sources for T2DM GWAS provide valuable information for researchers studying the genetics of T2DM. These data sources can be used to identify genetic variants associated with T2DM, to investigate the biological mechanisms.

**GWAS of Oily and non-oily fish intake by UK Biobank (UKB) consortium**^2^**.**

**Description (Oily fish):** Question asked: "How many servings of oily fish (e.g. salmon, tinned salmon, herring, mackerel, sardines, fresh tuna steak) did you have?" This question was only asked to participants who reported consuming fish or seafood.

**Description (Nonoily fish):** ACE touchscreen question "How often do you eat other types of fish? (e.g. cod, tinned tuna, haddock)" If the participant activated the Help button they were shown the message: Please provide an average considering your intake over the last year. If you are unsure, please provide an estimate or select Do not know.

This study provides a detailed analysis of the genomic analysis of dietary habits in UK Biobank, which identified hundreds of genetic associations. The study utilized complementary phenotyping approaches to complex dietary datasets and the utility of genomic analysis in understanding the relationships between diet and human health.

The GWAS on the 143 significantly heritable dietary habits, using linear mixed models in up to 449,210 individuals, identified 814 independent loci surpassing genome-wide significance. Of these, 309 also surpassed a more conservative Bonferroni-corrected study-wide significance threshold. Across the 143 dietary traits, there was a clear positive correlation of heritability estimates with the number of significant loci and the variance explained by these loci. The study found that education was positively associated with healthful dietary patterns, and that the identified olfactory receptor associations with fruit and tea intake may provide insight into the mechanisms underlying the health benefits of these foods. However, the study's Mendelian randomization approach had limitations in determining causality for coronary artery disease or type 2 diabetes.

The data sources for this study included UK Biobank, which provided the dietary data, and the Type 2 Diabetes Knowledge Portal, which made the GWAS results for the 143 significantly heritable dietary habits publicly available. All 170 derived dietary habits will be returned and shared through UK Biobank. Overall, this study provides valuable insights into the genetic associations of dietary habits and their impact on human health. The findings highlight the importance of considering both genetic and environmental factors in understanding the complex relationships between diet and health.

**GWAS of Low Density Lipoprotein Cholesterol and High Density Lipoprotein Cholesterol by** **Cristen J Willer *et al***^3^***.***

The GWAS study analyzed in this article aimed to identify new loci and refine known loci associated with lipid levels, including low-density lipoprotein (LDL) cholesterol, high-density lipoprotein (HDL) cholesterol, triglycerides, and total cholesterol. The study examined 188,578 individuals using genome-wide and custom genotyping arrays and identified and annotated 157 loci associated with lipid levels at P < 5×10^-8^, including 62 loci not previously associated with lipid levels in humans. Of the 62 novel loci, 24 demonstrated the strongest evidence of association with HDL cholesterol, 15 with LDL cholesterol, 8 with triglyceride levels, and 15 with total cholesterol. The effects of newly identified loci were generally smaller than in earlier GWAS. For the 62 newly identified variants, trait variance explained in the Framingham offspring were 1.6% for HDL cholesterol, 2.1% for triglycerides, 2.4% for LDL cholesterol, and 2.6% for total cholesterol .

To investigate connections between the new loci and known lipid biology, the study catalogued genes within 100 kb of the peak associated SNPs and searched PubMed and OMIM for occurrences of these gene names and their aliases in the context of relevant keywords. After manual curation, the study identified at least one strong candidate in 32 of the 62 loci (52%). For the remaining 30 loci, the study found no literature support for the role of a nearby gene on blood lipid levels . Among independent variants (r^2^ < 0.1) with P < 0.1 in the GWAS-only analysis, a significant excess were concordant in direction of effect for HDL (62.9% in 1,847 SNPs, P < 10^-16^), LDL (58.6% of 1,730 SNPs, P < 10^-16^), triglyceride levels (59.1% of 1,783 SNPs, P < 10^-16^), and total cholesterol (61.0% of 1,904 SNPs, P < 10^-16^), suggesting many additional loci to be discovered in future studies .

**GWAS of** **Body Mass Index (BMI) by** **GIANT consortium**^4^***.***

The summary statistics data sources for the genome-wide association study (GWAS) of Body Mass Index (BMI) were obtained from two previous studies: Wood et al. and Locke et al. Before conducting the meta-analysis with the UK Biobank (UKB) data, the researchers filtered out SNPs that did not match the pairs of alleles in the HRS and UKB and those that had reported allele frequencies that were too different from that calculated using unrelated participants of HRS. After filtering the data, the researchers performed a fixed-effect inverse variance weighted meta-analysis using the software METAL . This approach allowed them to combine the summary statistics from the two previous studies with the GWAS of height and BMI performed in ∼450 000 UK Biobank participants of European ancestry.

The combined GWAS meta-analysis reached N ∼700 000 individuals and substantially increased the number of GWAS signals associated with BMI. The researchers identified 941 near-independent SNPs associated with BMI at a revised genome-wide significance threshold of P < 1 × 10^-8^, including 751 BMI-associated SNPs located within loci not previously identified by these two GWAS. The near-independent genome-wide significant SNPs explained ∼6.0% of the variance of BMI in an independent sample from the Health and Retirement Study (HRS). This finding suggests that there are likely many more genetic factors that influence BMI that have yet to be identified.

To control for potential confounding variables, the researchers performed LDSC to quantify the level of confounding in GWAS due to population stratification as well as quantifying the genetic correlation between BMI and other traits. This approach allowed them to identify potential confounding variables and control for them in their analysis.

**GWAS of Glycated Hemoglobin A1c (HbA1c) by** **MAGIC**^5^***.***

Glycated Hemoglobin A1c (HbA1c) is a biomarker that reflects the average blood glucose levels over the past 2-3 months. It is widely used as a diagnostic tool for diabetes and as a monitoring tool for glycemic control in diabetic patients. In this summary statistics data source, we present the results of a genome-wide association study (GWAS) that aimed to identify common genetic variants associated with HbA1c levels in nondiabetic individuals of European ancestry.

The GWAS included up to 46,368 participants from 23 studies and 8 cohorts, and used de novo genotyped single nucleotide polymorphisms (SNPs) to test for associations with HbA1c levels. The study employed inverse-variance meta-analysis to combine the results of individual studies and tested mediation by glycemia using conditional analyses. The global effect of HbA1c loci was estimated using a multilocus risk score, and net reclassification was used to estimate genetic effects on diabetes screening. The results of the GWAS identified 10 loci that reached genome-wide significant association with HbA1c levels, including six new loci near FN3K, HFE, TMPRSS6, GCK, ANK1, and SPTA1. The lead SNP for FN3K was rs1046896 (P=1.6×10-^26^), for HFE was rs1800562 (P=2.6×10^-20^), for TMPRSS6 was rs855791 (P=2.2×10^-13^), for GCK was rs730497 (P=1.1×10^-10^), for ANK1 was rs4737009 (P=1.2×10^-9^), and for SPTA1 was rs2779116 (P=1.3×10^-9^). The other four loci were previously identified in other GWAS studies.

The study also found that the net reclassification of the population-level effect size of the seven nonglycemic HbA1c-associated SNPs was equivalent to reclassification of about 2% of a European ancestry population sample according to HbA1c-determined diabetes status. This suggests that the identified genetic variants have a modest effect on diabetes classification, but may still be useful for improving diabetes screening and diagnosis.

**GWAS of triglycerides (TG) by** **Richardson, Tom et al**^6^**.**

This study explored the following lipid metrics in the UK Biobank: low-density lipoprotein cholesterol (LDL-C), apolipoprotein B, triglycerides, high-density lipoprotein cholesterol (HDL-C), and apolipoproteins A-I. The lipid-related metrics in the UK Biobank were inverse rank-standardized/normalized so that they had a mean of 0 and a standard deviation of 1, which allowed for comparisons of the effect estimates between the different metrics .

GWAS analyses were conducted in UKBB participants of European descent based on K-means clustering (K = 4) after standard exclusions including withdrawn consent, mismatch between genetic and reported sex, and putative sex chromosome aneuploidy. We identified single nucleotide polymorphisms (SNPs) associated with each of the lipid-related traits using the BOLT-LMM (linear mixed model) software. Analyses were adjusted for age, sex, and a binary variable denoting the genotyping chip individuals were allocated to in UKBB (the UKBB Axiom array or the UK BiLEVE array). BOLT-LMM employs an LMM to evaluate the association between genetic variants and phenotypic traits whilst accounting for population stratification and cryptic relatedness. This approach has been shown to provide higher statistical power when applied to the approximately 459,000 European samples in the UKBB study compared to alternative approaches . Further details on genotyping quality control, phasing, imputation, and association testing have been reported previously. We assigned a SNP as associated with a lipid-related trait of interest through use of a conventional GWAS threshold (P < 5 × 10^-8^), and SNPs were binned into loci based on pairwise linkage disequilibrium (LD; at between-SNP r2 < 0.001), with the SNP with the strongest association with the trait of interest (as defined by P-value) being retained in each locus. This process (conventionally referred to as ‘LD clumping’) was undertaken for each trait in turn using the software PLINK, based on a reference panel of 503 Europeans from phase 3 (version 5) of the 1,000 Genomes Project. We defined novel SNPs as those associated with the trait of interest at P < 5 × 10^-8^ in our analyses in which an association had not been previously reported at P < 5 × 10^-8^, within 1 MB and at r2 < 0.001, by the Global Lipids Genetics Consortium (for LDL cholesterol, triglycerides, and HDL cholesterol) or by Kettunen and colleagues (for apolipoprotein B or apolipoprotein A-I).

**GWAS of C-Reactive protein (CRP) by** **Ligthart, S et al**^7^**.**

In this summary statistics data source, the authors present the results of a large-scale genome-wide association study (GWAS) of circulating amounts of CRP in over 350,000 individuals.

The GWAS was conducted using data from multiple cohorts, including both the HapMap and 1KG GWAS. The study design was pre-specified and included data quality checks, analysis, and data sharing. Serum CRP levels were measured using standard laboratory techniques, and individuals with autoimmune diseases, those taking immune-modulating agents, and those with CRP levels more than 4 standard deviations away from the mean were excluded from the analysis. The GWAS identified 3,977 genome-wide significant variants at p < 5 × 10^-8^, which mapped to 48 distinct loci. The authors used the proportion of CRP variance explained by the genetic instruments to perform power calculations for each outcome. They calculated the power to detect a relative 5%, 10%, 15%, and 20% difference in outcome risk.

In addition to the GWAS, the authors also conducted a large-scale cross-consortium Mendelian randomization study to investigate the causal relationship between CRP and 32 complex somatic and psychiatric outcomes. The study identified several causal relationships between CRP and various diseases, including coronary artery disease, rheumatoid arthritis, and schizophrenia. The authors also performed pathway analyses to identify biological pathways that are enriched for CRP-associated genes. They identified several pathways that are involved in immune function, including the complement system, cytokine signaling, and leukocyte migration.

**GWAS of insulin resistance (IR) by Dupuis J et al**^8^**.**

The GWAS of insulin resistance and related traits analyzed in this study included data from 21 cohorts, comprising up to 46,186 non-diabetic participants of European descent informative for fasting glucose (FG), and 20 GWAS including up to 38,238 non-diabetic individuals informative for fasting insulin (FI), as well as the surrogate estimates of β-cell function (HOMA-B) and insulin resistance (HOMA-IR) derived from fasting variables by homeostasis model assessment.

The study analyzed ~2.5 million directly genotyped or imputed autosomal SNPs from these cohorts, and identified 16 loci associated with fasting glucose and insulin resistance. Nine of these loci were novel, and five of the novel loci also demonstrated genome-wide significant evidence for association between the glucose-raising allele and T2D risk in up to 40,655 cases and 87,022 non-diabetic controls. The study found that HOMA-B and HOMA-IR have comparable heritability estimates, and their correlation is significant. However, the genetic architecture of each trait may be distinct, with more modest effects, fewer loci, rarer variants, or a stronger environmental modification underlying HOMA-IR. In addition, HOMA-IR is an imperfect estimate of global insulin resistance, as it addresses mostly hepatic sensitivity to insulin and is partially affected by β-cell function.

The study also identified likely biological candidate genes that influence signal transduction, cell proliferation, development, glucose-sensing, and circadian regulation within the identified loci. These genes provide new insights into the underlying biology of insulin resistance and related traits, and may reveal novel pathways for diabetes therapeutics.

**GWAS of 25-hydroxyvitamin D (25OHD) by Manousaki D et al**^9^**.**

The study used imputed genotypes from 401,460 white British UK Biobank participants with available 25OHD levels, retaining single-nucleotide polymorphisms (SNPs) with minor allele frequency (MAF) > 0.1% and imputation quality score > 0.3. The summary statistics data sources for this study include a Manhattan plot, a quantile-quantile (QQ) plot, and a list of 69 independent loci that contribute to serum 25OHD levels. The Manhattan plot displays the genome-wide association of 25OHD graphed by chromosome positions and -log10 P value. The QQ plot shows the distribution of observed P values compared to the expected distribution under the null hypothesis of no association.

The list of 69 independent loci that contribute to serum 25OHD levels was identified through a linear mixed model GWAS on standardized log-transformed 25OHD, adjusting for age, sex, season of measurement, and vitamin D supplementation. The loci were identified based on a threshold of genome-wide significance (P < 5 x 10^-8) and were independent of previously reported loci. In addition to the 69 independent loci, the study also identified 12 SNPs that achieved significant interaction p values. The direction of the beta for the interaction term genotype*season summer was in the same direction as the direction of the beta on 25OHD levels, meaning that the vitamin D lowering effect of these SNPs 'blunts' the expected increase in 25OHD in summer.

The study also conducted in silico functional follow-up gene prioritization and enrichment analyses. Gene prioritization analysis suggested 70 genes with false discovery rate (FDR) < 5% which might plausibly underlie the distribution of association statistics seen in the single variant results. At many loci, genes within the vitamin D metabolism pathway were suggested as plausible candidates. For example, DEPICT prioritized DHCR7 at the study.

| **Supplementary Table 1. Power calculations for bidirectional univariable Mendelian randomization analyses.** | | | | |
| --- | --- | --- | --- | --- |
| **Exposure** | **Outcome** | **Proportion of variance in the exposure explained by the instrument (*R^2^*)** | **F-statistic** | **Power-statistic** |
| Oily fish intake | T2DM | 0.45% | 45.56 | 100% |
| Non-oily fish intake | T2DM | 0.05% | 44.60 | 14% |
| T2DM | Oily fish intake | 27.29% | 75.56 | 100% |
| T2DM | Non-oily fish intake | 27.70% | 74.95 | 100% |
| **Meta-analysis** | | | | |
| Oily fish intake | T2DM (FinnGen) | 0.44% | 45.09 | 45% |
| Oily fish intake | T2DM (Xue A et al) | 0.31% | 46.74 | 95% |

| **Supplementary Table 2** Genetic Correlation Estimates for EA and Female reproductive traits or cancer by LDSC regression analysis | | | | |
| --- | --- | --- | --- | --- |
| **Exposure** | **Outcome** | **Genetic correlation(rg)** | **rg (SE)** | ***P*** |
| Oily fish intake | T2DM | -0.102 | 0.029 | 4.43E-04 |
| Non-oily fish intake | T2DM | 0.024 | 0.034 | 0.477 |
| **Phenotype** | **Heritability (h² )** | **h² (SE)** | **h² (Z)** | **h² (*P*)** |
| Oily fish intake | 5.76% | 0.003 | 19.041 | 7.75E-81 |
| Non-oily fish intake | 2.49% | 0.002 | 13.932 | 4.02E-44 |
| T2DM | 5.71% | 0.004 | 14.813 | 1.20E-49 |

| **Supplementary Table 3.** Summary of UVMR results | | | | | | | | | | | | | | |
| --- | --- | --- | --- | --- | --- | --- | --- | --- | --- | --- | --- | --- | --- | --- |
| **Exposure** | **Outcome** | **SNPs** | **IVW** | | |  | **MR Egger** | | |  | **Weighted median** | | | |
|  |  |  | **OR(95%CI)** | **P-value** | **PDR** |  | **OR(95%CI)** | **P-value** | **PDR** |  | **OR(95%CI)** | **P-value** | **PDR** |  |
| Oily fish intake | T2DM | 50 | 0.614 (0.504, 0.748) | 1.24×10^-6^ | 3.72×10^-6^ |  | 1.124 (0.501, 2.522) | 0.778 | 0.778 |  | 0.725 (0.583, 0.901) | 3.72×10^-3^ | 5.58×10^-3^ |  |
| Non-oily fish intake | T2DM | 9 | 0.902 (0.540, 1.505) | 0.692 | 0.695 |  | 1.656 (0.147, 18.639) | 0.695 | 0.695 |  | 0.842 (0.492, 1.440) | 0.529 | 0.695 |  |
| T2DM | Oily fish intake | 173 | 0.994 (0.988, 1.001) | 0.089 | 0.266 |  | 0.999 (0.984, 1.014) | 0.867 | 0.867 |  | 0.999 (0.989, 1.008) | 0.823 | 0.867 |  |
| T2DM | Non-oily fish intake | 177 | 1.001 (0.996, 1.006) | 0.765 | 0.949 |  | 1.001 (0.989, 1.012) | 0.949 | 0.949 |  | 1.004 (0.997, 1.012) | 0.275 | 0.824 |  |

| **Supplementary Table. 4** Summary of sensitivity results | | | | | | | | | | | | | | | | |
| --- | --- | --- | --- | --- | --- | --- | --- | --- | --- | --- | --- | --- | --- | --- | --- | --- |
| **Exposure** | **Outcome** | **MR-Egger intercept** | | |  | **MR-PRESSO global test** | | |  | **Cochrane’s Q** | | |  | **Steiger_test** | | |
|  |  | **Intercept** | **SE** | ***Pval*** |  | **RSS_obs_** | ***P*-value** | ***Outlier*** |  | ***Q*** | ***Q_df*** | ***Q_pval*** |  | **Direction** | ***Pval*** | **Filtered SNPs** |
| Oily fish intake | T2DM | -0.009224 | 0.0061 | 0.13 |  | 103.889 | *<0.0003* | *rs10061973,rs10510554,rs510161,rs59355765,rs6059844* |  | 99.99 | 49 | 2.37E-05 |  | TRUE | 6.24E-186 | NA |
| Non-oily fish intake | T2DM | -0.007791 | 0.0154 | 0.62 |  | 16.702 | *0.120* | *NA* |  | 13.89 | 8 | 0.0844 |  | TRUE | 7.36E-39 | NA |
| T2DM | Oily fish intake | -0.000327 | 0.0004 | 0.51 |  | 283.964 | <0.001 | *rs1061810,rs13130484,rs34990153* |  | 280.96 | 172 | 3.02E-07 |  | TRUE | 0 | NA |
| T2DM | Non-oily fish intake | 2.87E-05 | 0.0003 | 0.93 |  | 235.572 | *0.003* | *rs1061810,rs429358* |  | 233.21 | 176 | 0.002 |  | TRUE | 0 | NA |
| All results are after removing outliers and re-running the MR analysis | | | | | | | | | | | | | | | | |

**
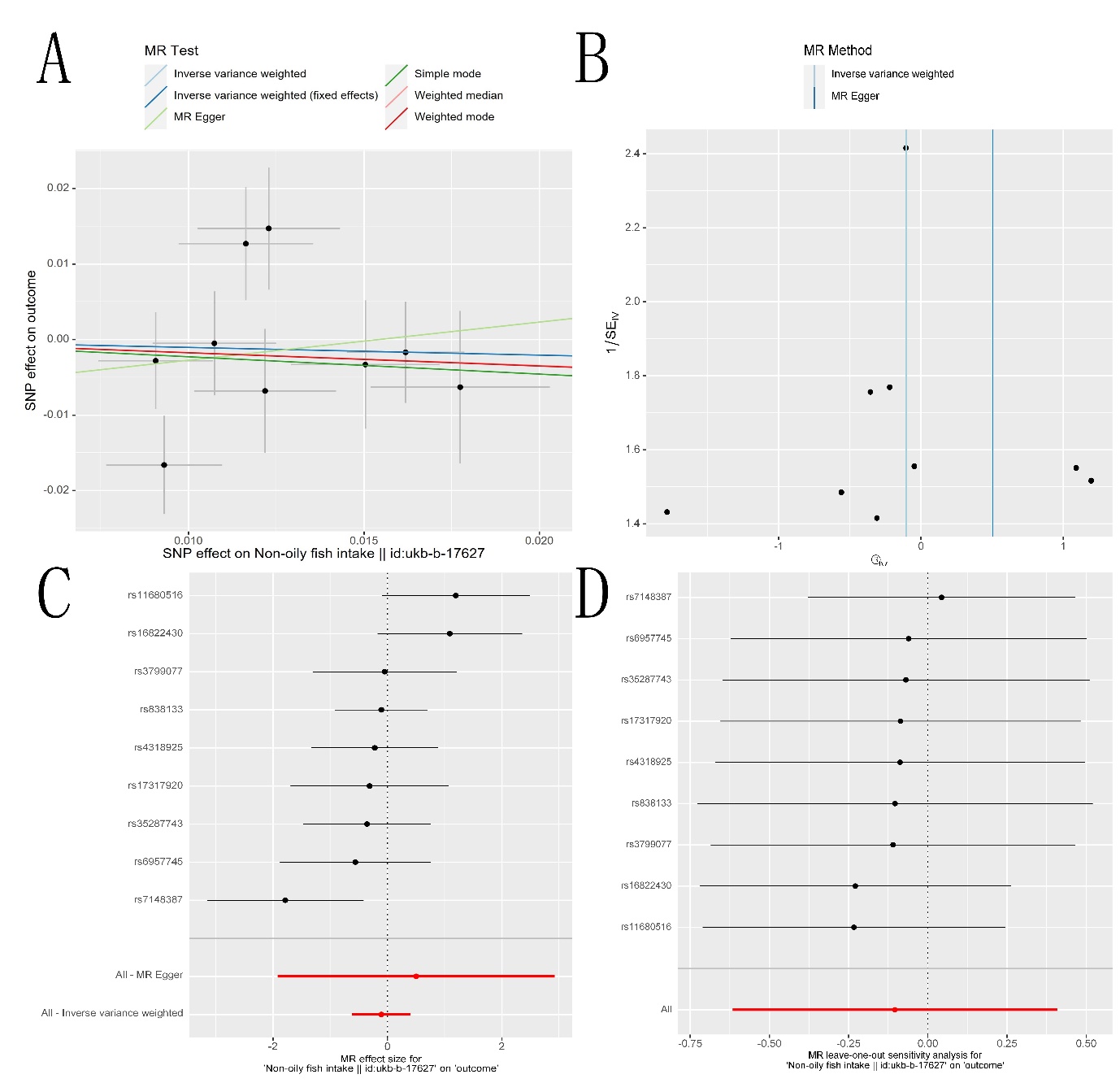
**

**Supplementary Fig. 1 Genetic associations with non-oily fish intake with T2DM at a genome-wide level of significance.** (A) Scatter plot. (B) Funnel plot. (C) Forest plots. (D) Leave-one-out plot. Horizontal and vertical lines represent 95% confidence intervals for the genetic associations. T2DM, T2DM, Type 2 Diabetes Mellitus.


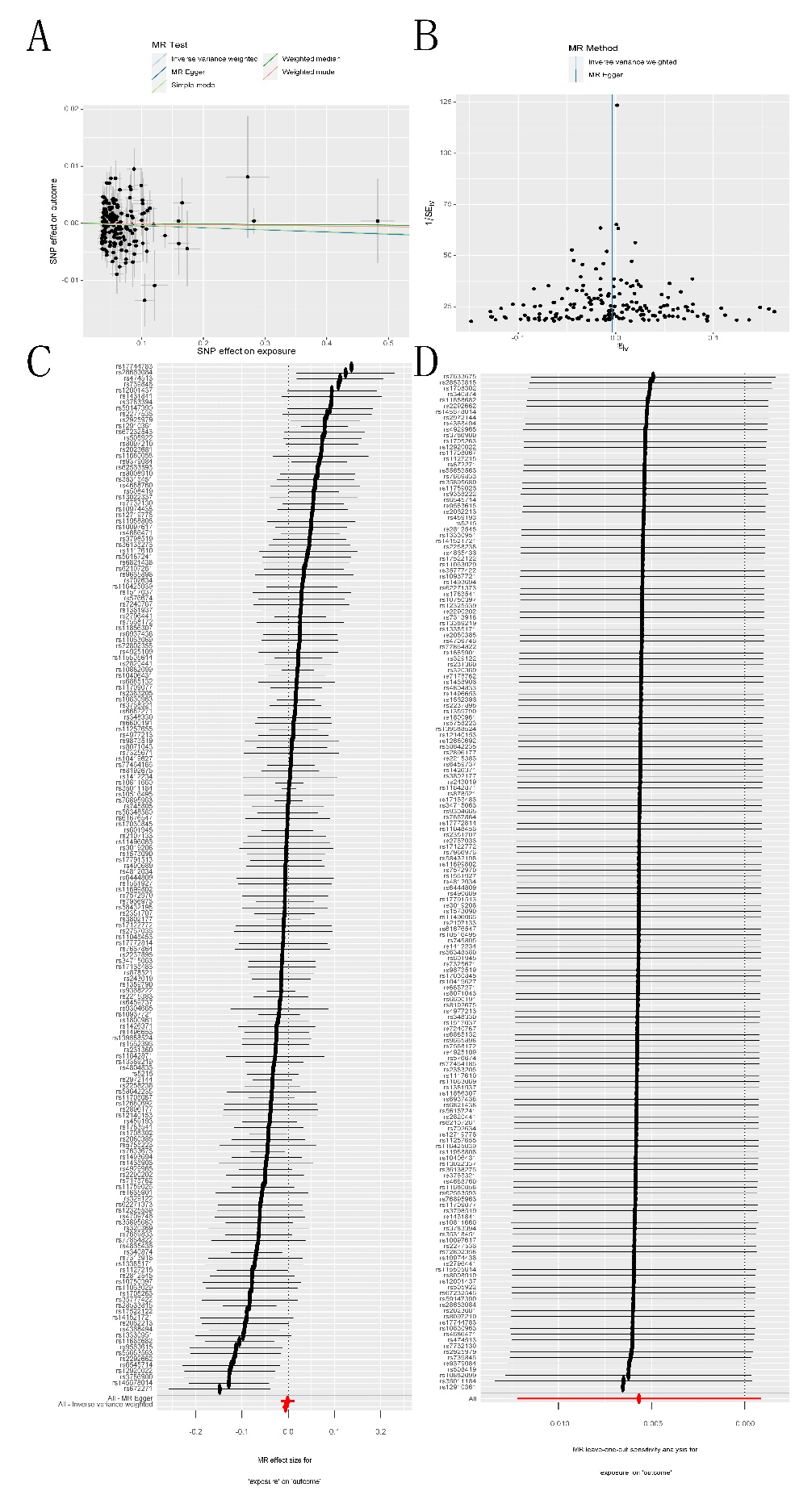


**Supplementary Fig. 3 Genetic associations with T2DM with oily fish intake at a genome-wide level of significance.** (A) Scatter plot. (B) Funnel plot. (C) Forest plots. (D) Leave-one-out plot. Horizontal and vertical lines represent 95% confidence intervals for the genetic associations. T2DM, T2DM, Type 2 Diabetes Mellitus.


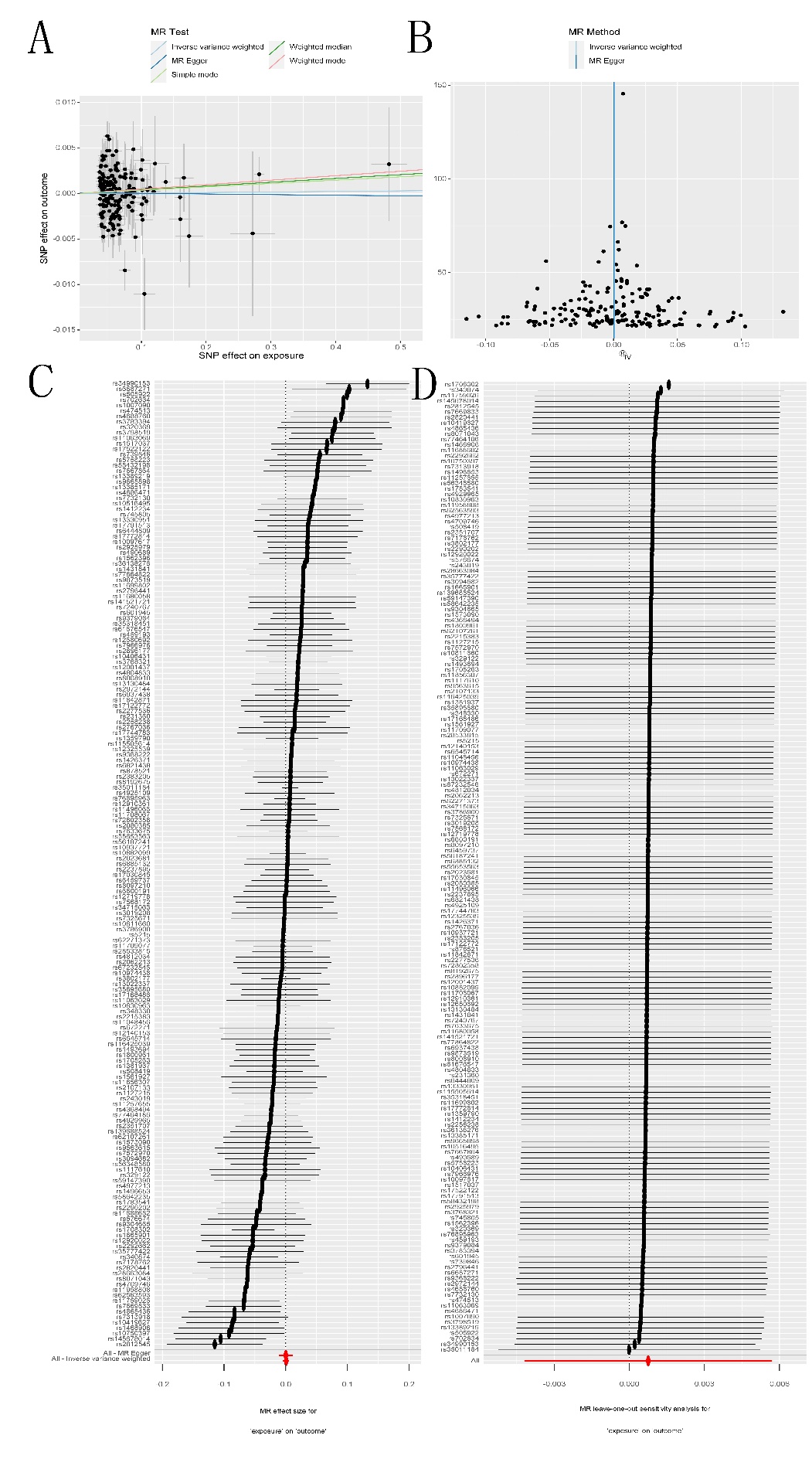


**Supplementary Fig. 4 Genetic associations with T2DM with non-oily fish intake at a genome-wide level of significance.** (A) Scatter plot. (B) Funnel plot. (C) Forest plots. (D) Leave-one-out plot. Horizontal and vertical lines represent 95% confidence intervals for the genetic associations. T2DM, T2DM, Type 2 Diabetes Mellitus.

**Reference**

1. Mahajan, A. *et al.* Multi-ancestry genetic study of type 2 diabetes highlights the power of diverse populations for discovery and translation. *Nat Genet* **54**, 560–572 (2022).

2. Cole, J. B., Florez, J. C. & Hirschhorn, J. N. Comprehensive genomic analysis of dietary habits in UK Biobank identifies hundreds of genetic associations. *Nat Commun* **11**, 1467 (2020).

3. Willer, C. J. *et al.* Discovery and refinement of loci associated with lipid levels. *Nat Genet* **45**, 1274–1283 (2013).

4. Yengo, L. *et al.* Meta-analysis of genome-wide association studies for height and body mass index in ∼700000 individuals of European ancestry. *Hum Mol Genet* **27**, 3641–3649 (2018).

5. Soranzo, N. *et al.* Common variants at 10 genomic loci influence hemoglobin A₁(C) levels via glycemic and nonglycemic pathways. *Diabetes* **59**, 3229–3239 (2010).

6. Richardson, T. G. *et al.* Evaluating the relationship between circulating lipoprotein lipids and apolipoproteins with risk of coronary heart disease: A multivariable Mendelian randomisation analysis. *PLoS Med* **17**, e1003062 (2020).

7. Ligthart, S. *et al.* Genome Analyses of >200,000 Individuals Identify 58 Loci for Chronic Inflammation and Highlight Pathways that Link Inflammation and Complex Disorders. *Am J Hum Genet* **103**, 691–706 (2018).

8. Dupuis, J. *et al.* New genetic loci implicated in fasting glucose homeostasis and their impact on type 2 diabetes risk. *Nat Genet* **42**, 105–116 (2010).

9. Manousaki, D. *et al.* Genome-wide Association Study for Vitamin D Levels Reveals 69 Independent Loci. *Am J Hum Genet* **106**, 327–337 (2020).
